# Diesel Exhaust Particles Activate the Matrix-Metalloproteinase-1 Gene in Human Bronchial Epithelia in a β-Arrestin–Dependent Manner via Activation of RAS

**DOI:** 10.1289/ehp.0800311

**Published:** 2008-10-29

**Authors:** Jinju Li, Andrew J. Ghio, Seung-Hyun Cho, Constance E. Brinckerhoff, Sidney A. Simon, Wolfgang Liedtke

**Affiliations:** 1 Department of Neurobiology, Duke University, Durham, North Carolina, USA;; 2 U.S. Environmental Protection Agency, University of North Carolina, Chapel Hill, North Carolina, USA;; 3 U.S. Environmental Protection Agency, Research Triangle Park, Research Triangle Park, North Carolina, USA;; 4 Departments of Medicine and Biochemistry, Norris Cotton Cancer Center, Dartmouth-Hitchcock Medical Center, Lebanon, New Hampshire, USA;; 5 Center for Neuroengineering and; 6 Division of Neurology, Department of Medicine, Duke University, Durham, North Carolina, USA

**Keywords:** β-arrestin, bronchial epithelia, diesel particles, MAP kinase, MMP-1, *MMP-1* promoter polymorphism, urban smog

## Abstract

**Background:**

Diesel exhaust particles (DEPs) are globally relevant air pollutants that exert a detrimental human health impact. However, mechanisms of damage by DEP exposure to human respiratory health and human susceptibility factors are only partially known. Matrix metalloproteinase-1 (MMP-1) has been implied as an (etio)pathogenic factor in human lung and airway diseases such as emphysema, chronic obstructive pulmonary disease, chronic asthma, tuberculosis, and bronchial carcinoma and has been reported to be regulated by DEPs.

**Objective:**

We elucidated the molecular mechanisms of DEPs’ up-regulation of *MMP-1*.

**Methods/Results:**

Using permanent and primary human bronchial epithelial (HBE) cells at air–liquid interface, we show that DEPs activate the human *MMP-1* gene via RAS and subsequent activation of RAF-MEK-ERK1/2 mitogen-activated protein kinase signaling, which can be scaffolded by β-arrestins. Short interfering RNA mediated β-arrestin1/2 knockout eliminated formation, subsequent nuclear trafficking of phosphorylated ERK1/2, and resulting *MMP-1* transcriptional activation. Transcriptional regulation of the human *MMP-1* promoter was strongly influenced by the presence of the –1607GG polymorphism, present in 60–80% of humans, which led to striking up-regulation of *MMP-1* transcriptional activation.

**Conclusion:**

Our results confirm up-regulation of *MMP-1* in response to DEPs in HBE and provide new mechanistic insight into how these epithelia, the first line of protection against environmental insults, up-regulate *MMP-1* in response to DEP inhalation. These mechanisms include a role for the human –1607GG polymorphism as a susceptibility factor for an accentuated response, which critically depends on the ability of β-arrestin1/2 to generate scaffolding and nuclear trafficking of phosphorylated ERK1/2.

The production of diesel exhaust particles (DEPs) by vehicular traffic is a major contributor to urban particulate matter air pollution (McClellan 1987; McClellan et al. 1985; Sydbom et al. 2001; Torres-Duque et al. 2008). Inhalation of diesel exhaust is associated with cardiovascular diseases (e.g., atherosclerosis, arrhythmias, thrombosis) and respiratory diseases [e.g., chronic asthma, chronic obstructive pulmonary disease (COPD), bronchial cancer], leading to an increase in mortality (Bayram et al. 2006). DEPs form aggregates approximately 0.1–0.5 μm in diameter that can penetrate into more distal branches of the bronchial tree. Because of the large number of hazardous chemicals that are present on DEPs, their pathologic effects on airways and lungs are pleiotropic, as documented in numerous studies that have focused on various pathologic mechanisms. Specifically, DEPs have been shown to increase the secretion of proinflammatory cytokines, release phosphatidylcholine, produce reactive oxygen species that lead to oxidative injury, and induce DNA damage, any or all of which may compromise lung function (Bayram et al. 2006; Cao et al. 2007a; Danielsen et al. 2008; Ghio et al. 2000; Madden et al. 2000; Nikula et al. 1995; Singh et al. 2004; Zhang et al. 2004).

Matrix metalloproteinase-1 [MMP-1; Ensembl Gene ID ENSG00000196611 (Ensembl 2008)] is a zinc-dependent endo-peptidase that has been shown to exert detrimental effects on respiratory health. MMP-1 is secreted from cells as an inactive precursor of the active proteinase, zymogen (Pardo and Selman 2005). MMP-1 plays a role in tissue remodeling and repair during development, in inflammation, and in the invasion, migration, and metastasis of malignantly transformed cells (Boire et al. 2005; Ishii et al. 2003). A polymorphism in the *MMP-1* 5′-regulatory region –1607G(G) exerts a powerful effect on transcriptional activation, and the 1607GG sequence forms an Ets transcription-factor binding site, which acts as a transcriptional activator (Brinckerhoff and Matrisian 2002; Rutter et al. 1998; Tower et al. 2002).

Activation of *MMP-1* has been shown to be of great relevance for airway and lung health and disease. MMP-1 is involved in airway extra-cellular matrix degradation and alveolar wall stability and is pathogenetically linked to both malignant and nonmalignant chronic respiratory diseases (Elkington et al. 2005; Mercer et al. 2004, 2006; National Heart, Lung, and Blood Institute 2007; Segura-Valdez et al. 2000), including COPD, chronic asthma, emphysema, lung tuberculosis, and bronchial carcinoma.

Two studies have examined the putative role of DEP-induced *MMP-1* activation in lung cells. Doornaert et al. (2003) reported a decrease in MMP-1 expression when HBE cells (16HBE14o-) were exposed to DEPs. In contrast, Amara et al. (2007) investigated the effects of DEPs on MMP-1 expression in A549 and NCI-H292 lung epithelial tumor cell lines and found it increased and dependent on the NADP(H) oxidase/NOX4 redox-dependent mechanism. Given these seemingly conflicting results and the relevance of increased MMP-1 expression for human respiratory health, we addressed this issue in permanent and primary human bronchial epithelial (HBE) cells, the latter assayed at air–liquid interface, using a DEP preparation high in organic content realistically generated by diesel engines in cars, trucks, buses, locomotives, and boats (Bechtold et al. 1985; Hirano et al. 2003).

We found that DEPs led to increased activation of *MMP-1* in BEAS-2B bronchial epithelia and primary HBE cells that was linked to specific activation of RAS, which leads to activation of RAF-MEK-ERK1/2 signaling. Signaling was fully dependent on scaffolding by both β-arrestin isoforms, enabling mitogen-activated protein (MAP) kinase signaling, which activates *MMP-1* in the nucleus via phosphorylated extracellular signal-regulated kinase (phospho-ERK1/2). We also found that the regulatory effect of DEPs on the *MMP-1* gene critically involved the –1607GG *MMP-1* promoter polymorphism that is present in 25% of Caucasians homozygously, and 50% heterozygously, and with similar frequencies in Asian and African-American populations (Fujimoto et al. 2002). Thus, in most humans, breathing DEP-polluted air may trigger increased *MMP-1* activation in airway epithelia, making them vulnerable to chronic airway and lung injury.

## Material and Methods

### Cell culture and DEPs

#### BEAS-2B human airway epithelial cells

We obtained BEAS-2B cells, which are SV-40 adenovirus–transformed immortalized bronchial epithelial cells (Agopyan et al. 2003; Ghio and Cohen 2005; Ke et al. 1988), from American Type Culture Collection (Rockville, MD). We used cells between passages 65 and 85. Cells were maintained in keratinocyte growth media (Clonetics, San Diego, CA) supplemented with bovine pituitary extract (0.22–1.54 mg/ mL total lipid), human epidermal growth factor (5 ng/mL), hydrocortisone (0.5 mg/ mL), ethanolamine (0.1 mM), phospho-ethanolamine (0.1 mM), and insulin (5 mg/ mL). All supplemental tissue culture reagents were purchased from Sigma (St. Louis, MO). For imaging studies, 1 day before use, cells were plated at a density of 1 × 10^5^ cells/mL on 12-mm-diameter poly-D-lysine–coated glass coverslips (Carolina Biological Supply, Burlington, NC).

#### Primary HBE cells

All investigations with primary human cells were approved by the institutional review board of the EPA and Duke University; all studies complied with all applicable requirements of the United States and customary international regulations. All participants provided written informed consent before the study. We obtained HBE cells from healthy, nonsmoking adult volunteers by cytologic brushing of the airways during bronchoscopy (Ghio and Cohen 2005). We grew these cells to passage 3 in bronchial epithelial growth medium (BEGM) (Clonetics, San Diego, CA), plated them on 12-well culture plates with collagen-coated filters with a 0.4-μm pore size (Trans-CLR; Costar, Cambridge, MA) at a density of 1 × 10^5^ cells/filter. The cells were maintained in a 1:1 mixture of BEGM and HEPES-supplemented Dulbecco’s modified Eagle’s medium with singlequot supplements, bovine pituitary extracts (13 mg/mL), bovine serum albumin (1.5 mg/mL), and nystatin (20 U/mL) in 0.5 mL in the apical chamber and 1.5 mL in the basal chamber. Fresh medium was provided every 48 hr. Media was removed from the apical chamber at least 24 hr before use to create an air–liquid interface (Ghio and Cohen 2005). For measurement of secreted MMP-1, we sampled media from the lower compartment. Cells were used 3–5 days after passage 3; that is, they did not yet display a terminally differentiated phenotype (Turi et al. 2006). Compared with BEAS-2B permanent cells, primary HBE cells maintained in air–liquid interface culture devices had a 5- to 10-fold increased cell density, resulting in 5- to 10-fold increased absolute number of cells per assay.

### DEPs and P90 control carbon nanoparticles

DEPs were the generous gift of D. Costa and I. Gilmour at the U.S. Environmental Protection Agency (EPA) in Research Triangle Park, NC (Singh et al. 2004). The DEPs were generated at the U.S. EPA main campus in Research Triangle Park (NC, USA) using a 30-kW four-cylinder Deutz BF4M1008 diesel engine connected to a 22.3 kW Saylor Bell air compressor to provide load. The emissions from the engine were diluted with filtered air (3:1), the temperature adjusted to approximately 35°C, and the emission directed to a small baghouse (Dustex model T6-3.5-9 150 ACFM with nine polyester felt bags). The emissions were collected by a conventional pulsing system (reverse air pulsing). While the baghouse was pulse-cleaned, DEPs filled the bottom of the baghouse in a conical section. After 45 min, the contents were emptied and immediately refrigerated. In order to simulate a more “realistic” environmental condition, “EPA DEP MIX” was generated by mixing DEPs collected at three different engine operations. The engine and compressor were operated at steady state using approximately 25% of the engine’s rated full load. The organic:elemental carbon ratio of DEP MIX was estimated to be 0.47, calculated from the weight percentage of each DEP in the DEP MIX and the measured organic:elemental carbon ratio of each filter sample collected from the different engine operations using a thermal-optical carbon analyzer.

Degussa Printex 90 (P90) carbon nano-spheres were the generous gift of W. Moller (GSF National Research Center for Environment and Health, Neuherberg/Munich, Germany) (Moller et al. 2005). Investigations at GSF have demonstrated that P90 nanoparticles comprise relatively “clean” carbon particles, that is, very low in organic contamination and low in metal/water-soluble contamination. In view of this, and because their size is similar to the DEP carbonaceous cores, we used P90 nano-particles as control particles.

We applied DEPs and P90 nanoparticles in concentrations between 10 and 100 μg/mL, subjected to rigorous vortexing (30 sec) before application.

### Chemicals/pharmacologics

Pharmacologic inhibitors and targeted pathways included PD98059 and UO126 to specifically inhibit MEK MAP kinase, SB203580 for inhibition of p38 MAP kinase, SP600125 for inhibition of JNK MAP kinase, and LBT613 and AAL881 to inhibit RAF (Hjelmeland et al. 2007; Sathornsumetee et al. 2006). All chemicals were purchased from Tocris (Ellisville, MO), except the RAF inhibitors, which were a generous gift of D. Batt, (Novartis, Cambridge, MA). We chose the concentrations based on recommendations of the supplier (Tocris) or those commonly used in previous studies (Ahn et al. 2003; Walker et al. 2003), usually 5–10 μM, except for LBT613, which we used at 1 μM (Hjelmeland et al. 2007).

### Probing gene expression of MMP-1 by real-time quantitative reverse transcriptase polymerase chain reaction

We quantified relative gene expression of *MMP-1* in human airway epithelial cells using real-time quantitative reverse transcriptase polymerase chain reaction (qRT-PCR). Total RNA was isolated using the RNAeasy kit (Qiagen, Valencia, CA) and reverse transcribed to generate oligo-dT–primed cDNA. *MMP-1* primer/probe sets were obtained as Taqman predeveloped assay reagents [concentrated and preoptimized mix of primers and Taqman probe labeled with 6-carboxy-fluorescein (FAM)] from Applied Biosystems (Foster City, CA).Quantitative fluorogenic amplification of cDNA was performed using the ABI Prism 7500 Sequence Detection System (Applied Biosystems), *MMP-1* primer/probe sets of interest, and TaqMan Universal PCR Master Mix (Applied Biosystems). We determined the relative abundance of mRNA levels from standard curves generated from a serially diluted standard pool of cDNA prepared from cultured human airway epithelial cells. The relative abundance of βactin mRNA was used to normalize levels of-*MMP-1* mRNAs.

### Transfection of MMP-1–promoter reporter constructs

The human *MMP-1* reporter plasmids −4400, −3292, −2942, −2002, −1546, and −517 used in this study harbored the firefly luciferase (fLUC) reporter gene under the transcriptional control of the *MMP-1* promoter (Mercer et al. 2006; Rutter et al. 1998; Tower et al. 2003). 1 μg DNA from promoter constructs was transiently transfected using ExGen 500 (Fermentas, Glen Burnie, MD) into BEAS-2B cells and plated in 24-well plates. As evidenced by fluorescent reporters, transfection efficiency was > 70% (data not shown). After transfection, cells were washed and incubated for 24 hr with or without DEPs and/or chemical modulators. Cell lysates were generated using 25 mM Tris (pH 7.8), 2 mM EDTA, 10% glycerol, 2 mM dithiothreitol, and 1% Triton X-100 and were subsequently assayed for luciferase activity using the Dual Luciferase Reporter Assay System (Promega, Thousand Oaks, CA), in a 96-well-plate luminometer (Veritas Microplate Luminometer, Turner Biosystems, Mountain View, CA). All transfections were carried out in triplicate or in quadruplicate, and cells were cotransfected with a promoterless *Renilla* luciferase construct to control for transfection efficiency and toxicity/viability. Data were normalized by *Renilla* activity and presented as mean ± SE.

### Immunocytochemistry

For immunofluorescence studies, we preincubated sections for 30 min at room temperature in phosphate-buffered saline (PBS) containing 0.3% Triton X-100 (PBS-Triton) and 10% normal donkey serum. The sections were then incubated for 24 hr at 4°C with mouse monoclonal MMP-1–specific antibody (MAB901; R&D Systems, Minneapolis, MN) that was diluted in PBS-Triton and incubated with fluorescein isothiocyanate–conjugated donkey anti-mouse IgG (H+L; Jackson Immunoresearch, Philadelphia, PA). Fluorescent micrographs were recorded using a BX61 Olympus microscope equipped with the respective filter sets or a Leica SP2 confocal laser scanning platform. Control experiments were conducted using identical amounts of nonspecific isotype mouse antibody (R&D Systems).

### Enzyme-linked immunosorbent assays and MMP-1 cleavage activity assay

**Figure 1:**
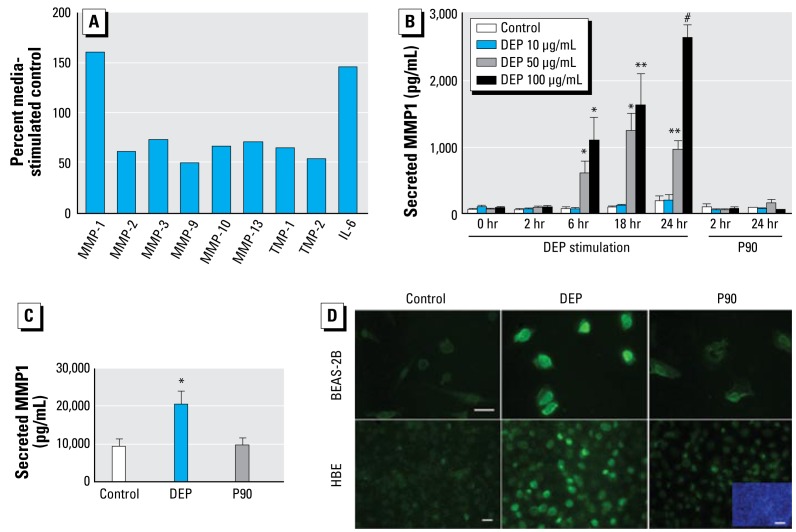
Active MMP-1 is secreted from HBE in response to DEPs. (*A*) Results of MMP and cytokine screen of DEP-stimulated BEAS-2B cell supernatant: secretory responses of several MMPs, TIMPs, and IL-6 (positive control). Cells were stimulated in triplicate (50 μg/mL DEPs; supernatants pooled after 6 hr). (*B*) After exposure to increasing concentrations of DEPs, secretion of MMP-1 by BEAS-2B cells followed a time course and a dose–response pattern by ELISA. P90 nanoparticles served as control; experiment conducted six times. (*C*) In primary HBE, DEP (100 μg/mL) exposure evoked robust MMP-1 secretion by ELISA. Note also the high amount of unstimulated MMP-1 secretion. P90 nanoparticles served as control; experiment conducted in triplicate. (*D*) MMP-1 immunofluorescence (BEAS-2B, primary HBE cells) identifies cell-bound MMP-1 immunoreactivity (green). In BEAS-2B cells, stimulation with P90 nanoparticles does not surpass background; DEP stimulation (100 μg/mL) leads to robust up-regulation, uniformly staining all cells. Specificity of the DEP MMP-1 response could also be observed in primary HBE cells, yet here a more heterogeneous MMP-1 immunoreactivity is apparent. 4’-6’-diamidino-2-phenylindole (DAPI) nuclear stain illustrates increased cell density (blue insert in the lower right-hand micrograph). Scale bars = 20 μm for MMP-1 immunolabeling, 80 μm for the DAPI. *p < 0.05, **p < 0.01 , and ^#^*p* < 0.001 compared with controls.

We initially conducted a cytokine multiplex assay (BioPlex Hu-27-Plex; Bio-Rad Laboratories, Hercules, CA) and a simplified matrix-metalloproteinase proteomics array (SearchLight Human MMP Arrays 1 and 2; Endogen-Pierce, Rockford, IL). In agreement with previous studies (Bayram et al. 1998), we found interleukin-6 (IL-6) to be significantly up-regulated after DEP exposure (Figure 1A), thus assuring the validity of our cytokine multiplex assay.

We performed MMP-1 enzyme-linked immunosorbent assay (ELISA) following standard protocols (Kim et al. 2005; Peake et al. 2005). Briefly, to measure MMP-1 in cell culture supernatant of BEAS-2B cells and in the lower compartment of primary airway epithelia kept at air–liquid interface, Nunc MaxiSorp ELISA plates (eBioscience, Termecula, CA) were coated overnight at 4°C with MAB901 (R&D Systems). Nonspecific binding to plates was blocked with 1% bovine serum albumin in PBS for 1 hr at room temperature. After washing the cells with PBS/0.05% Tween 20, culture supernatants/fluids and the MMP-1 standard dilutions were added to wells for overnight incubation at 4°C. After washing, detection antibody (goat anti-human MMP-1, R&D Systems) was added for 90 min at room temperature, followed by washing and donkey anti-goat IgG horseradish peroxidase–conjugated detection antibody for 45 min. A chromogenic ELISA substrate (K-blue, Sigma) was then added for color development, which was arrested with 1 M H_2_SO_4_. Plates were read at 450 nm in an ELISA plate reader using SoftmaxPro software (Molecular Devices, Sunnyvale, CA), which led directly to determination of MMP-1 concentrations in samples.

MMP-1 cleavage activity was measured using a commercially available assay (SensolytePlus 520; Anaspec, San Jose, CA). In this assay, MMP-1 is captured by immobilized MMP-1 antibodies, and its proteolytic activity is measured by a 5-FAM/QXLTM520 peptide that evokes fluorescent resonance energy transfer (FRET), bearing a specific MMP-1 cleavage site. The fluorescence of 5-FAM, which acts as fluorophore, is quenched by QXLTM520, which acts as a quencher in the intact FRET peptide. Upon specific cleavage by active MMP-1, the fluorescence of 5-FAM is recovered, and the dual emission of 490/520 nm is monitored.

None of the colorimetric readouts were impaired by the use of DEPs as stimulus (data not shown).

### Western blotting

We performed Western blotting using standard methods (Cao et al. 2007). Briefly, cells were lysed with radio-immunoprecipitation assay (RIPA) buffer, separated on an 10% sodium dodecyl sulfate (SDS) polyacrylamide gel, and then transferred to polyvinylidene fluoride (PVDF) filters (0.45 μm pore; Millipore, Amherst, MA) by semidry blot using Tris-glycine/20% methanol transfer buffer. Blotted proteins were immuno-detected using a two-step antibody-mediated chemiluminescence assay using specific antibodies against MMP-1 (1:1,000; R&D Systems), MEK, ERK1/2, their phosphorylated isoforms (1:500 to 1:2,000; Cell Signaling Technology, Danvers, MA), and secondary peroxidase-coupled antibodies (1:5,000; Jackson Immunoresearch).

### Trafficking assay of phospho-ERK

After stimulation with DEPs, we fixed cells in 4% paraformaldehyde at 0, 10, 20, 30, and 60-min time points. Cells were then immunolabeled for phospho-ERK1/2, using a mouse monoclonal phospho-ERK1/2–specific antibody (Cell Signaling Technology), which was immunodetected by standard secondary reagents (Molecular Probes, Invitrogen, Carlsbad, CA), using an upright fluorescent microscope (Olympus BX61) or a laser confocal scanning microscope (Leica SP2). Nuclear abundance of phospho-ERK1/2 was determined densitometrically on micrographs recorded at respective time points with fixed image acquisition settings. ImageJ freeware was used for analysis (National Institutes of Health 2008) and averaged data were plotted against time.

### β-Arrestin short interfering RNA experiments

These experiments used established protocols (Ahn et al. 2003; DeWire et al. 2007). Briefly, chemically synthesized, double- stranded short interfering RNAs (siRNAs) were purchased from Dharmacon Research (Lafayette, CO). We derived the sequences from Ahn et al. (2003). The siRNA sequences targeting β-arrestin-1 (GenBank accession no. NM_020251; National Center for Biotechnology Information 2008) and β-arrestin-2 (NM_004313) are 5′-AAAGCCUUCUG-CGCGGAGAAU-3′ and 5′-AAGGACCGC AAAGUGUUUGUG-3′ and correspond to positions 439–459 and 201–221, respectively, relative to the start codon. Another RNA duplex was synthesized, used as a control (5′-AAGUGGACCCUGUAGAUGGCG- 3′; position 101–120 from the start codon of β-arrestin-1, sequence common to both arrestins), and found to have no silencing effects on β-arrestin -1 or -2 expression (Ahn et al. 2003; DeWire et al. 2007). BEAS-2B cells were then transfected with siRNA using Lipofectamine transfection reagent (Invitrogen) following established guidelines (Rippmann et al. 2005). Forty-eight hours after transfection, cells were divided into 24-well plates for *MMP-1*–fLUC reporter gene assays and MMP-1 ELISA of the supernatant. All assays were performed in triplicates, with two independent experiments. For cellular immunodetection of β-arrestin, we used a mouse monoclonal antibody that recognizes both arrestin isoforms (BD Bioscience, San Diego CA), and for β-arrestin Western blot detection, an antibody generously provided by R. Lefkowitz (Duke University) raised against a common region in the C-terminus.

### Transfection of dominant-negative RAS

We obtained the dominant-negative RAS mutant N116Y as a fusion to enhanced green fluorescent protein (eGFP) in pcDNA3.1 plasmid as a generous gift from R. Yasuda (Duke University) (Watanabe et al. 2000; Yasuda et al. 2006). This plasmid (500 ng/well in a 24-well plate) was transfected into BEAS-2B cells using ExGen500 DNA transfection reagent (Fermentas) according to manufacturer instructions.

### Statistical analysis

Mean and SEs of quantified outcome parameters after DEP stimulation were compared with their respective controls. For experiments involving modulation of the response, we likewise compared the increases from stimulation with DEPs alone versus DEPs plus inhibitor. Group comparisons were accomplished using fixed-effect one-way analysis of variance (ANOVA) with post hoc Scheffe test for multigroup comparisons. Minimum significance was set at the 0.05 level.

## Results

### DEPs increase MMP-1 secretion in human permanent and primary bronchial epithelia

As a starting point of our investigation, we used commercially available multiplex ELISAs to screen the supernatant of DEP-exposed BEAS-2B airway epithelial cells for up-regulation of proinflammatory mediators. MMP-1 emerged as the mediator with the strongest up-regulation among MMPs. We also found IL-6 to be up-regulated, which was previously reported for airway cells in response to DEPs (Steerenberg et al. 1998), thus validating our findings. In addition, there was a slight down-regulation of several other MMPs as well as their endogenous inhibitors, tissue inhibitor of metalloproteinase-1 (TIMP-1) and TIMP-2 (Figure 1A). MMP-1 secretion was increased by DEPs as a function of time (2–24 hr) and dose (10–100 μg/mL), but not by a comparable concentration of P90 nanoparticles that served as inert carbon control particles (Figure 1B). These findings agree with a recent report of MMP-1 up-regulation (Amara et al. 2007) but not with the other study reporting conflicting results (Doornaert et al. 2003). MMP-1 is also strongly up-regulated in primary HBE cells assayed at an air–liquid interface (Figure 1C, D). Western blotting of BEAS-2B cells confirms this result [see Supplemental Material, Figure 1 (http://www.ehponline.org/members/2008/0800311/suppl.pdf)]. Using immunocytochemistry, we found that HBE cells exhibit a more heterogeneous expression pattern of MMP-1 than do BEAS-2B cells (Figure 1D). Density of MMP-1–expressing cells was higher in HBE than in BEAS-2B cells. Nevertheless, we noticed that in unstimulated conditions, HBE cells secrete significantly more MMP-1 than do BEAS-2B cells, exceeding a factor 10 to account for increased cell density of HBE cells. Finally, we demonstrated that secreted protein correlates with specific MMP-1 cleavage activity [see Supplemental Material, Figure 2 (http://www.ehponline.org/members/2008/0800311/suppl.pdf)].

### DEPs increase MMP-1 transcription in an allele-specific manner

**Figure 2:**
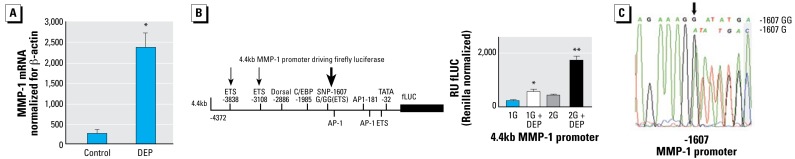
DEP MMP-1 response increases in an allele-specific manner for the human–1607G(G) *MMP-1* promoter polymorphism. (*A*) In BEAS-2B cells exposed to DEPs (100 μg/mL), *MMP-1* mRNA formation is increased (vs. P90 nanoparticle control) as evidenced by relative abundance of the *MMP-1* transcript, determined by Taqman real-time qRT-PCR. Statistically significant up-regulation of *MMP-1* mRNA; experiment conducted in triplicate. (*B*) A 4.4-kb *MMP-1* promoter reporter gene construct was transfected into BEAS-2B cells. Schematic illustrates the promoter with transcription factor binding sites (left). The large arrow denotes the –1607G(G) human polymorphism, which generates an ETS transcription factor binding site for GG; two smaller arrows denote additional upstream ETS binding sites. Diagram at right illustrates *MMP-1*–fLUC reporter gene activity (normalized for *Renilla*), in relative units (RU). Note that in response to DEPs (100 μg/mL; 24-hr incubation; triplicate assays; data based on three or more experiments), fLUC activity was strikingly increased for –1607GG (2G). (*C*) DNA sequencing of the *MMP-1* promoter, encompassing –1607, from BEAS-2B cells. Like approximately 50% of all humans, BEAS-2B cells have a heterozygous genotype (–1607GG; –1607G). In (*A*), *p < 0.05 up-regulation of *MMP-1*. In (*B*), *p < 0.05, and **p < 0.01, significant difference compared with the unstimulated condition.

The dose dependency and time course of secreted MMP-1 protein in response to DEPs suggest transcriptional regulation of the *MMP-1* gene. Therefore, we investigated transcriptional regulation of *MMP-1* in response to DEPs, and the possible role of the human 1607G(G) polymorphism. Increased transcription of *MMP-1* was confirmed by qRT-PCR in BEAS-2B cells stimulated with DEP versus P90 nanoparticles (100 μg/mL, 24-hr time point; *n* = 3 independently stimulated dishes per group, Taqman real-time qRT-PCR methodology; Figure 2A; results consistent with Amara et al. 2007). To examine whether increased *MMP-1* mRNA abundance in response to DEPs is due to increased mRNA stability or to increased transcription, we conducted luciferase reporter gene assays using the 4.4-kb *MMP-1* promoter. We employed both isoforms of the promoter, –1607G and –1607GG, to elucidate the impact of the promoter polymorphism on gene regulation. Figure 2B shows that up-regulation of *MMP-1* transcription causes increased *MMP-1* mRNA abundance. Also, the –1607GG polymorphism potentiated *MMP-1* transcription. Figure 2C shows the genotype of BEAS-2B cells to be heterozygous –1607GG; –1607G, indicating that transcription of the endogenous *MMP-1* gene in these cells is carried by both alleles.

A recent study on transcriptional regulation of *MMP-1* in response to cigarette smoke extract, a complex mix of noxious, pro-oxidative chemicals/irritants (in this respect similar to DEPs), reported a distal “tobacco-response element” from −2.9 to −4.4-kb of the *MMP-1* promoter. In view of this, we transfected a set of luciferase reporters harboring *MMP-1* promoters of varying length [see Supplemental Material, Figure 3 (http://www.ehponline.org/members/2008/0800311/suppl.pdf)] into BEAS-2B cells and stimulated them with DEPs. The 2.9-kb promoter (–1607GG) exhibited the highest activity, followed by 3.3-kb and 4.4-kb promoters. This suggests the presence of sequences that function to bind transcriptional repressors, in response to DEPs, within the “tobacco-response element” (−2.9 to −4.4 kb). This regulation was appreciable for the −1607GG polymorphism. For shorter constructs, iterative reduction of the 2.9-kb promoter decreased transcriptional activation.

### DEPs specifically activate the RAS-RAF-MEK-ERK1/2 MAP kinase pathway to activate MMP-1

Activation of the MMP-1 gene has been shown to be critically linked to mitogen-activated protein kinase activation (Cao et al. 2007; Hecht et al. 2007; Pillinger et al. 2007), and involvement of MEK-ERK was recently reported for DEP-stimulated lung tumor cells, depending on NADPH oxidase (Amara et al. 2007). We used BEAS-2B bronchial epithelial cells to explore whether MAP kinases function as intracellular signal transducers leading to transcriptional activation of *MMP-1*. This was accomplished by inhibiting MEK, JNK, and p38 MAP kinases with specific antagonists; RAF with novel specific inhibitors, and RAS using a dominant-negative genetic construct. We used inhibitory compounds on BEAS-2B and primary HBE cells, whereas we used the RAS dominant-negative gene construct only in BEAS-2B cells.

**Figure 3:**
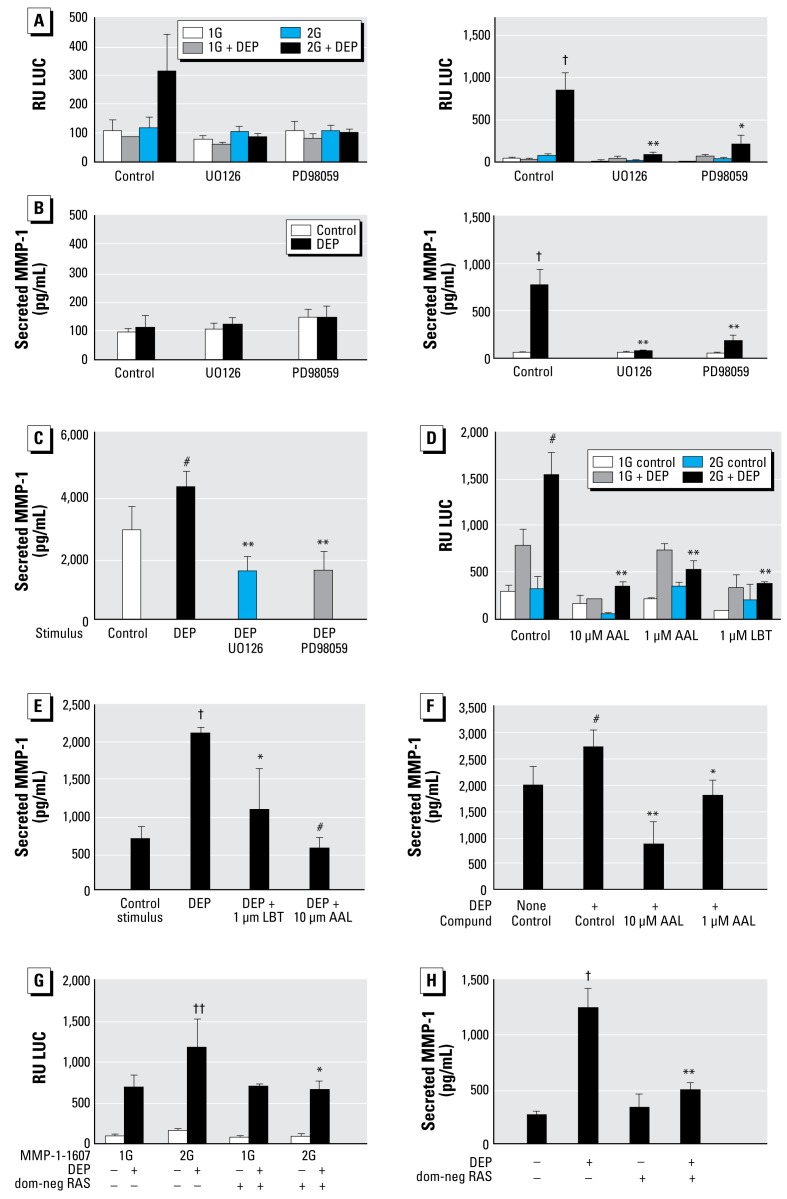
MAP kinase signaling via the RAS-RAF-MEK-ERK1/2 pathway is necessary for the DEP MMP-1 response in HBE. (A–C) MEK inhibition. (*A* and *B*) Dependence of the DEP MMP-1 response on MEK (and ERK1/2): transcriptional activation of *MMP-1* at 2 hr (*A* ; left) and 24 hr (*A*; right) in relative units (RU), and secretion of MMP-1 at the same time points (*B*). Both responses depend on MAP kinase signaling by MEK-ERK1/2, evidenced by the effect of UO126 and PD98059 (both 10 μM). (A and B; right) For transcription, differences reach statistical significance for the −1607GG *MMP-1* polymorphism (A; right) For secretion of MMP-1, differences caused by MEK inhibitors are statistically signifi-cant. DEP stimulation was with 100 μg/mL; experiments conducted in triplicate with two independent experiments. (*C*) Effect of MEK inhibitors UO126 and PD98059 on primary HBE cells. Note the complete prevention of MMP-1 secretion. Experiment carried out in quadruplicate, with 100 μg/mL DEPs. (*D–F*) RAF inhibition with AAL881 and LBT613 down-regulated transcription of *MMP-1* fLUC reporter genes. (*D*) Down-regulation strongest for the −1607GG polymorphism. (*E* ) Secretion of MMP-1 protein from BEAS-2B cells. (*F*) Secretion of MMP-1 protein from primary airway epithelia. Experiments conducted in triplicate, with at least two independent experiments, using 100 μg/mL DEPs. (*G* and *H*) RAS inhibition [transient transfection of a dominant-negative (dom-neg) RAS] down-regulated the DEP MMP-1 response. (*G* ) Transcriptional activation of *MMP-1* and secretion of MMP-1 after 24 hr measured using *MMP-1*–fLUC assays (4.4-kb *MMP-1* promoter, –1607G and –1607GG). Cells transfected with the –1607G polymorphism showed no effect; further reduction of transcription for the –1607GG polymorphism was statistically significant, yet incomplete. (*H* ) Transcriptional activation of *MMP-1* and secretion of MMP-1 after 24 hr measured using MMP-1 ELISA. Transfection of dominant-negative RAS without DEP stimulation had no effect on *MMP-1* transcription. Dominant-negative RAS virtually eliminated MMP-1. Experiment conducted in triplicate with two sets of independent experiments. *p < 0.05, **p < 0.01, and ^#^*p* < 0.001, indicate significant inhibition of the increase caused by DEP. ^##^*p* < 0.05, *^†^p* < 0.01, and *^††^p* < 0.001 indicate statistically significant differences from control.

Specific inhibitors of MEK (UO126, PD98059) down-regulated transcription (Figure 3A), an effect that was more accentuated for the –1607GG polymorphism. In keeping with the –1607G;–1607GG genotype of BEAS-2B cells, secretion of MMP-1 was also profoundly down-regulated in these cells (Figure 3B). To examine whether primary HBE cells show an identical activation pattern of MAP kinases, we inhibited MEK in primary HBE cells, which also led to a decrease in MMP-1 secretion. MEK inhibition led to even lower levels of secreted MMP-1 compared with nonstimulated, nontreated cells, pointing toward a “tonic drive” along the MEK-ERK MAP kinase pathway in these cells (Figure 3C). We next examined whether RAF functions up-stream of MEK. Inhibition of RAF using the novel specific inhibitors LBT613 and AAL881 was as effective as MEK inhibition (Figure 3D–F). Based on this finding, we examined whether RAF is activated by the membrane bound GTPase RAS. In order to inhibit RAS, the dominant-negative RAS isoform was transfected heterologously into BEAS-2B cells, which led to a marked down-regulation of the MMP-1 response (Figure 3G, H). However, taking into account an estimated 60–70% efficiency for transient transfection of BEAS-2B cells, as indicated by transfection of fluorescent reporter genes (data not shown), a partial down-regulation of the MMP-1 response indicates a powerful impact of dominant-negative RAS on downstream RAF signaling. Regarding the specificity of these findings, inhibition of JNK with SP600125 and p38 with SB203580 did not significantly (*p* > 0.3) down-regulate transcription of *MMP-1* or MMP-1 secretion [see Supplemental Material, Figure 4 (http://www.ehponline.org/members/2008/0800311/suppl.pdf)]. The fourth known MAP kinase pathway, involving ERK5, was also tested using phospho-ERK5 Western blot, and it was not activated by DEPs (data not shown). Thus, we conclude that activation of RAS, which leads to RAF-MEK-ERK1/2 MAP kinase signaling, but not p38, JNK, or ERK5, is selectively implicated in *MMP-1* activation.

### MMP-1 up-regulation depends on both isoforms of β-arrestin

**Figure 4:**
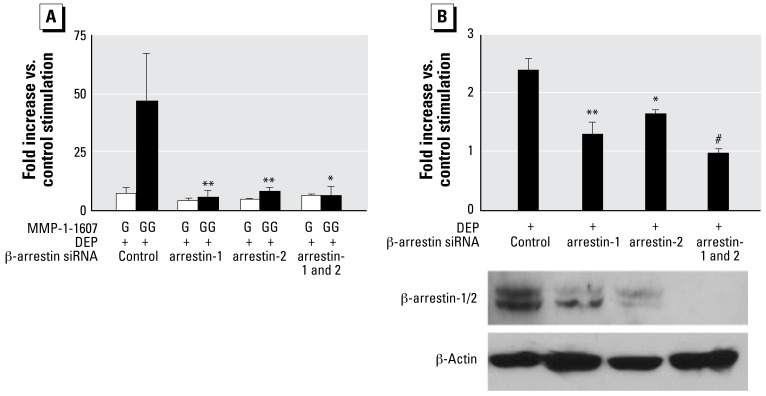
siRNA-mediated knockdown of both β-arrestins eliminates the DEP MMP-1 response. (*A*) Up-regulation of *MMP-1* transcription using luciferase reporter genes (4.4-kb promoter, –1607G and –1607GG) in response to DEPs (100 μg/mL, 24 hr) and its striking down-regulation after knocking down β-arrestin-1 and -2 either separately or in combination. Fold increase for the –1607G promoter (vs. control stimulation) was considerably weaker and not statistically significantly different. On the other hand, the large increase for the –1607GG promoter was robustly reduced for β-arrestin knockdown, with β-arrestin-2 exhibiting a slightly weaker effect than β-arrestin-1 and a complete elimination for combined knockout. siRNA was transfected 72 hr before DEP stimulation. Experiment conducted in triplicate, with two independent experiments. (*B*) Fold increase of MMP-1 secretion (BEAS-2B cells) after stimulation with DEPs (100 μg/mL), and the respective effect of knockdown of β-arrestins. Knockdown for β-arrestin-1 lowers the MMP-1 response significantly and is stronger than knockdown of β-arrestin-2, with the highest impact with both arrestins combined. Western blots show the effect of knockdown of both β-arrestins on their respective protein abundance (normalized for β-actin). Pan-arrestin siRNA leads to complete β-arrestin knockout. siRNA was transfected 72 hr before DEP stimulation. Experiment conducted in triplicate, with two independent experiments. *p < 0.05, **p < 0.01, and ^#^*p* < 0.001 compared with fold increase obtained for DEP stimulation using control siRNA.

We next examined the role of β-arrestins, given their known association with RAF-MEK-ERK1/2 signaling (Ahn et al. 2003; Dasgupta et al. 2006; DeWire et al. 2007; Lohse et al. 1990; McDonald et al. 2000). In recognition of the emerging role of β-arrestins as scaffolding proteins that bind directly to RAF and MEK and indirectly to ERK1/2, orchestrating their function in a “signalosome” (Naumann et al. 1999), we investigated the function of β-arrestins in the DEP MMP-1 response by employing a previously reported β-arrestin–specific siRNA (Ahn et al. 2003; DeWire et al. 2007; Lohse et al. 1990; McDonald et al. 2000). We used BEAS-2B cells because they have been successfully subjected to siRNA gene knockdown (Cao et al. 2007a; Rippmann et al. 2005). Knocking down β-arrestins led to a down-regulation of the targeted proteins and resulted in a down-regulation of *MMP-1* transcription and MMP-1 secretion (Figure 4A,B). MMP-1 down-regulatory effects of a specific knockdown for β-arrestin-1 were more noticeable than for β-arrestin-2 (Figure 4A,B), yet both were statistically significantly different from control (*p* < 0.01 for protein secretion and transcriptional activation). However, the most complete effect was obtained using combined β-arrestin -1 and -2 targeting, which amounted to a protein knockout for both β-arrestins, as revealed by Western blot (Figure 4B) and led to complete elimination of transcription and secretion of *MMP-1* and MMP-1. These striking effects clearly demonstrate that, in response to DEPs, both β-arrestins are necessary for mediating *MMP-1* activation.

DEP-evoked formation of phospho-ERK1/2 and its subsequent transfer to the nucleus depends on RAS-RAF-MEK-ERK1/2 MAP kinase signaling and β-arrestins. β-Arrestins’ function as scaffolds for RAF-MEK-ERK1/2 has been shown to encompass retention of phospho-ERK in the cytoplasm (Ahn et al. 2003; DeWire et al. 2007), yet in another study, they were associated with nuclear translocation of phospho-ERK (Kobayashi et al. 2005; Gesty-Palmer et al. 2005). As a concept, activation of *MMP-1* via phospho-ERK is understood to involve nuclear translocation of the latter (Roberts and Der 2007; Tower et al. 2002). In order to resolve this issue, we established a time course for nuclear translocation of phospho-ERK1/2, using immunocytochemistry for phospho-ERK1/2 after stimulation with DEPs.

**Figure 5:**
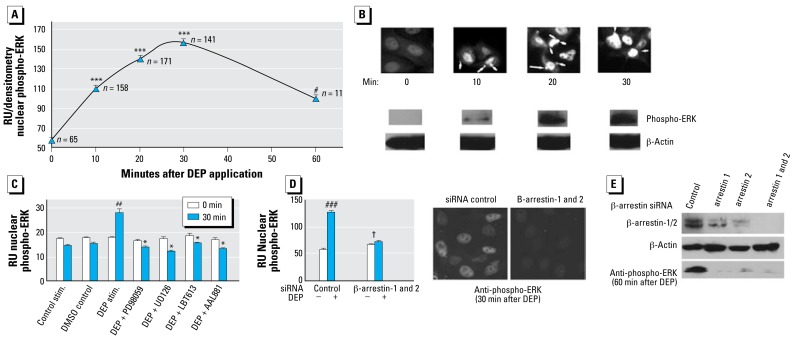
Phospho-ERK1/2 is trafficked to the nucleus as an early signaling event of the DEP MMP-1 response. (*A*) Time course of nuclear phospho-ERK1/2 after stimulation of BEAS-2B cells with DEPs (100 μg/mL). Nuclear phospho-ERK was detected by immunofluorescence and its abundance was evaluated by densitometry of the nucleus (cell numbers indicated beneath each coordinate). (*B*) Representative micrographs after DEP exposure of nuclear phospho-ERK immunofluorescence. Arrows indicate DEPs in direct contact with a cell. Western blots below show increased abundance of phospho-ERK in whole-cell lysate. (*C*) Nuclear phospho-ERK1/2 at 0 min and 30 min. A significant increase for DEP stimulation (100 μg/mL) was absent in controls (media, 0.2% DMSO). Chemical inhibition of MEK and RAF eliminated generation of a nuclear signal for phospho-ERK (MEK: UO126, PD98059, both used at 10 μM; RAF: 1 μM LBT613 and 10 μM AAL881). Eighty cells were analyzed per condition after DEP stimulation, and 40 before stimulation. (*D*) Knockdown of β-arrestins also eliminated generation of a nuclear signal for phospho-ERK. The graph (left) shows quantitation of control siRNA versus anti–pan-arrestin siRNA. The confocal micrographs (right) show representative findings. Eighty cells were analyzed for each condition after DEP stimulation, and 40 before stimulation. (*E*) Western blot as shown in (*C*) plus the corresponding Western blot for whole-cell phospho-ERK. Cell lysates sampled at 60 min. ***p < 0.001 difference from time point 0 min. ^#^*p* < 0.01 difference from time point 30 min. ^##^ *p* < 0.01 compared with control. *p < 0.01 reduction of the 0–30 min difference obtained when stimulating with DEPs. *^###^* *p* < 0.01 difference between DEP-stimulated and control-stimulated. *^†^p* < 0.001 down-regulation.

A time course of phospho-ERK1/2, using immunofluorescent labeling, indicated that phospho-ERK enters the nuclear compartment as early as 10 min, with a peak at the 30 min time point (Figure 5A,B), confirmed by confocal laser scanning microscopy [see Supplemental Material, Figure 5 (http://www.ehponline.org/members/2008/0800311/suppl.pdf)]. These findings are not in keeping with the concept of cytoplasmic retention of phospho-ERK1/2 by β-arrestins, but rather with previously reported experiments that demonstrate β-arrestin–dependent nuclear translocation (Kobayashi et al. 2005). Our data clearly suggest a logical temporal sequence that involves early entry of phospho-ERK1/2 into the nuclear compartment, leading to early transcriptional activation of *MMP-1*. These data and their interpretation are in good agreement with our 2-hr time point findings for activation of the *MMP-1* fLUC-reporter gene (Figure 3A). Using the presence of nuclear phospho-ERK1/2 at 30 min after stimulation as a read-out of the effect of DEPs, we found no increase of nuclear phospho-ERK1/2 when chemically inhibiting RAF and MEK and when knocking out both isoforms of β-arrestin (Figure 5C–E). The confocal image of β-arrestin knockout cells shows a lack of phospho-ERK1/2 staining both in nucleus and cytoplasm, and a virtual elimination of whole-cell phospho-ERK1/2 when using Western blotting, with total ERK constant. Thus, both β-arrestins are necessary for formation of phospho-ERK1/2. Without phospho-ERK1/2, there is no nuclear translocation and subsequent regulation of *MMP-1* in response to DEPs.

## Discussion

**Figure 6:**
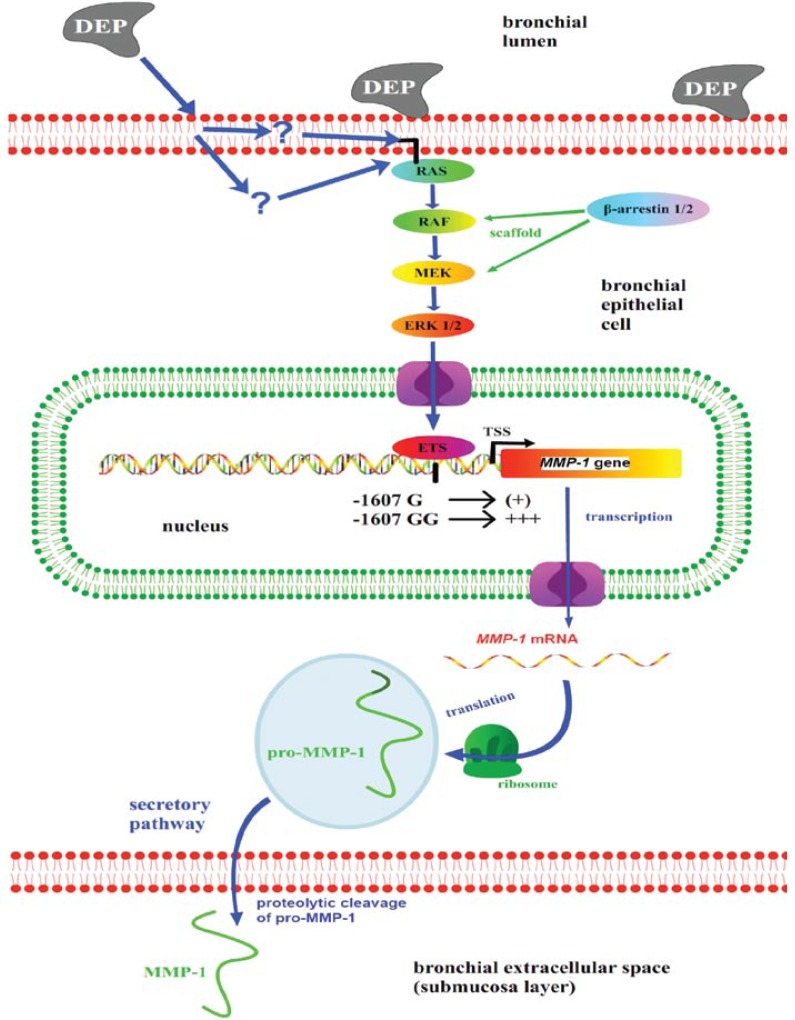
Proposed model of DEP-mediated increased expression of MMP-1 by HBE. DEPs lead to activation of the membrane-bound GTPase RAS, which in turn switches on the MEK-ERK1/2 MAP kinase via RAF signaling. Phospho-ERK is translocated to the nucleus, where it activates the *MMP-1* gene [transcription start site (TSS)]. ETS binding sites in the *MMP-1* promoter are significant for this activation, in particular, the presence of such a site at position –1607, –1607GG, a known human susceptibility locus for lung and airway disease. *MMP-1* mRNA is translated, and MMP-1 is secreted into the abluminal bronchial submu-cosal layer. Although proven for several cell types and stimuli, scaffolding for RAF and MEK by β-arrestins via direct binding awaits experimental proof.

Using human airway epithelial cells, we have shown that DEPs lead to increased transcriptional activation of the *MMP-1* gene and subsequent secretion of MMP-1. This mechanism is powerfully boosted by the–1607GG polymorphism within the *MMP-1* promoter, which is present in at least one allele in approximately 75% of humans and forms a known ETS transcription factor binding site (Rutter et al. 1998). Intracellular constituents that carry this signal transduction are the MEK-ERK1/2 MAP kinase cascade, with necessary upstream activation of RAF and RAS (see schematic overview in Figure 6). RAF-MEK-ERK1/2 MAP kinase signaling is known to be scaffolded by β-arrestins-1 and -2. Accordingly, we found that both β-arrestins were necessary for formation of phospho-ERK1/2, its subsequent trafficking to the nucleus, and transcription of *MMP-1*. Thus, our findings are suggestive of a mechanism for this activation. Because MMP-1 has been linked to both nonmalignant and malignant respiratory disorders, results presented here increase our understanding of how airborne DEPs can injure bronchial epithelia, sensitize airway sensory nerve afferents, and thus damage human airways and lungs in a context of several highly relevant respiratory disorders e.g., emphysema, COPD, chronic asthma, lung tuberculosis, and bronchial carcinoma (Kim et al. 2004; Mercer et al. 2004; Rutter et al. 1998; Torres-Duque et al. 2008).

Two previous investigations reported on the phenomenon of DEPs regulating *MMP-1* expression: one reported a decrease (Doornaert et al. 2003), and the other an increase (Amara et al. 2007). We differ from the study reporting a MMP-1 decrease, a discrepancy that could possibly be related to particle, cell, or MMP-1 ELISA technology. Our results are consistent with the other study showing DEPs increased MMP-1 activation based on an NADP(H) oxidase–dependent pathway (for the relevance of DEPs evoking oxidant-mediated injury, see Amara et al. 2007; Bayram et al. 2006; Cao et al. 2007b; Ghio et al. 2000; Madden et al. 2000; Zhang et al. 2004; for using tumor-derived alveolar cells, see Amara et al. 2007). Here, we have used bronchial epithelial cells, both permanent and primary, with the latter assayed at air–liquid interface. Of note, studies on the regulation of the human *MMP-1* gene cannot readily be complemented by studies in mice (or rats), because rodents do not have a valid ortholog of this gene (Brinckerhoff and Matrisian 2002). In addition, our DEP preparation contained an increased concentration of organic components compared with the DEPs used in the study with tumor-derived alveolar cells (Amara et al. 2007), thus providing a stimulus more realistically reflecting the exhausts from road, rail, and ship traffic.

Three particular aspects of the intracellular signaling mechanisms leading to activation of *MMP-1* deserve comment. First, we have shown that the –1607GG *MMP-1* polymorphism in the 5′ regulatory region of the human *MMP-1* gene exerts a dominant influence on *MMP-1* transcription and is more effective than the –1607G promoter. From a public health perspective, this is highly relevant because, regardless of ethnicity, at least one copy of the –1607GG allele is present in 60–80% of humans (Fujimoto et al. 2002). This finding suggests an enhanced susceptibility to the detrimental effects of DEP inhalation, possibly together with tobacco smoking (Mercer et al. 2004, 2006, 2008; Mercer and D’Armiento 2006; Torres-Duque et al. 2008), which leads to activation of *MMP-1*, in most people that populate habitats with significant DEP exposure (Fujimoto et al. 2002).

Second, we have extended our mechanistic understanding of the role of the MEK-ERK1/2 pathway in up-regulating the *MMP-1* gene in response to DEPs, first reported by Boczkowski’s group (Amara et al. 2007). For the first time, we demonstrate an essential role for RAF and RAS in this signaling. We have identified the necessity of MEK-ERK1/2 in primary HBE and shown its critical influence on transcriptional activation of the *MMP-1* gene that is potentiated by the –1607GG allele. In addition, we have shown that after DEP exposure, within 30 min, newly generated phospho-ERK relocates to the nucleus.

Intracellular signaling cascades do not simply operate in a diffusely solubilized environment within the cytoplasm, but are functionally tightly organized by scaffolding proteins, which constitute specific “signalosomes” for any signaling cascade that functions in response to a specific stimulus (DeWire et al. 2007; Gesty-Palmer et al. 2005). In this respect, our third contribution is that the cellular response to DEPs is dependent on cell-growth–related MAP kinases that are dependent on both β-arrestins. In contrast to the well-documented concept of β-arrestin-mediated scaffolding of MAP kinase signaling with cytoplasmic retention of phospho-ERK (Ahn et al. 2003; DeWire et al. 2007), we have observed nuclear translocation of phospho-ERK in the presence of β-arrestin -1 and -2, in agreement with a recent study that reported nuclear translocation of phospho-ERK dependent on β-arrestin-2 in immortalized cultured cells transfected with the β_2_ adrenoceptor (Kobayashi et al. 2005). Our finding that both β-arrestins are closely linked to this MAP kinase pathway has not been previously reported. Mechanistically, in the absence of both β-arrestins, phospho-ERK1/2 was not generated (Figure 5D,E). The study by Dasgupta et al. (2006) serves as an interesting comparison. They examined the role of β-arrestin-1–mediated scaffolding in lung tumor cells in response to nicotine, with the outcome of cell growth. siRNA-mediated knockdown of β-arrestin-1 led to nonformation of phospho-ERK in response to nicotine, with subsequent elimination of the growth response. β-Arrestin-1 was recruited to the nicotinergic acetylcholine receptor, which was instrumental for downstream signaling leading to activation of RAF. β-Arrestins might function similarly in bronchial epithelia exposed to DEPs, but *a*) both β-arrestins were necessary for the DEP MMP-1 response, not specifically one of them; *b*) cell lines in the study of Dasgupta et al. (2006) were derived from bronchial tumors because the focus of their study was cell growth of such tumors in response to nicotine, a single compound that has a specific cognate cell surface receptor; and *c*) Dasgupta et al. (2006) provided evidence of a functional and dynamic protein–protein interaction of the nicotinergic acetylcholine receptor with β-arrestin-1, but based on their data, an additional role for β-arrestin-1 in scaffolding RAF-MEK cannot be excluded.

Regarding the potential human health issues associated with this study, RAS-mediated MAP kinase signaling is a growth-related pathway also known to be dysfunctionally activated in malignant transformation of tumors (Roberts and Der 2007; Sridhar et al. 2005). In this context, we note that pathogenesis of bronchial cancer is also linked to extracellular-matrix–degrading properties of MMP-1, which includes tumor cell growth, invasion, and metastatic capability (Pritchard et al. 2001; Rutter et al. 1998). Our findings in cultured airway epithelia can thus be viewed as a reflection of the well-known epidemiologic association between breathing polluted, DEP-containing urban smog and increased incidence of bronchial cancer in humans (Hemminki and Pershagen 1994; McClellan et al. 1985; Parent et al. 2007). The findings from this study position us favorably to ask whether known cancer risk cofactors, namely, cigarette smoking and genetic predisposition, enhance *MMP-1* activation in combination with DEP exposure. With respect to cigarette smoking, smoke components can enhance *MMP-1* transcription via the –1607GG polymorphism. In the study by Mercer et al. (2008), the distal 1-kb of the same 4.4-kb promoter that we have used here proved to be essential for activation of the *MMP-1* gene by cigarette smoke extract, a “chemicotoxicologic library” of several thousand compounds, in this respect similar to DEP-bound chemicals. As in our study, there was increased GG-allele stimulus-responsive activity. Remarkably in contrast, however, the “tobacco-responsive elements” from −2.9 to −4.4kb were critical for an increase in activity in response to cigarette smoke extract, whereas our results point to a strikingly repressive mode of action [see Supplemental Material, Figure 3 (http://www.ehponline.org/members/2008/0800311/suppl.pdf)]. When Mercer et al. (2008) conducted deletion, bioinformatics, and transcription-factor binding studies on the distal segment of the 4.4-kb *MMP-1* promoter, they found PEA3 transcription factors acting as robust repressors, with binding sites at a “tandem” position (−3838 and −3824 relative to transcriptional start site). PEA3 and its respective tandem binding sites are attractive candidates to function as repressors of *MMP-1* transcription in the response to DEPs. However, when comparing the response profile of the different reporter gene constructs of Mercer et al.’s study with ours, it is apparent that detailed transcriptional regulation of *MMP-1* reveals different mechanisms, dependent on the airway-injury–inducing stimulus. Regarding genetic factors as lung cancer risk factors, 30% of all bronchial carcinomas have mutations in RAS that render its activity more or even constitutively active (Roberts and Der 2007; Sridhar et al. 2005).

In summary, we have elucidated signaling mechanisms operative in HBE and how the disease-enhancing *MMP-1* gene is activated in response to DEPs. We provide evidence that in primary HBE cells the growth-related MAP kinase signaling pathway is critical for DEP-evoked up-regulation of *MMP-1* and subsequent secretion of MMP-1. We also present, for the first time, data that this regulation depends on activation of RAS and RAF as well as both isoforms of β-arrestin, which we found necessary for formation of phospho-ERK1/2. In the presence of both β-arrestin isoforms, phospho-ERK1/2 translocates to the nucleus, peaking at 30 min. This cascade of events leads to transcriptional activation of *MMP-1* that is significantly more robust for the –1607GG *MMP-1* promoter polymorphism, present in most humans.

In terms of translational medical implications, our findings suggest the potential for topical delivery of compounds to human airways that can down-regulate RAS-induced MAP kinase signaling, culminating in the activation of *MMP-1*, which might be of benefit for patients at risk of developing ultimately devastating respiratory diseases linked to *MMP-1* dysregulation (Mercer and D’Armiento 2006). Such topical applications, in order to inhibit MAP kinase signaling, have also been proposed recently for cystic fibrosis patients (Boncoeur et al. 2008; Roque et al. 2008).
